# Surgical decompression strategies for treatment of multilevel cervical myelopathy comparison of outcome with single level cases

**Published:** 2012-11

**Authors:** Misagh Shafizad, Guive Sharifi

**Affiliations:** ^*a*^Neurosurgery Department, Loghman Hakim Hospital, Shaheed Beheshti University of Medical Sciences, Tehran, Iran.

## Abstract

**Background:**

Selecting the treatment of choice for multilevel cervical myelopathy remains a controversial issue. The most important factor in choosing a treatment method for patients with cervical myelopathy is the sagittal curvature and the site of compressive pathology.

**Methods:**

A total of 20 cases were available for the last 4 years with acceptable follow-up (f/u) of at least one year after surgery. Clinical examinations were obtained before surgery, 3 months post-surgery and at f/u. Evaluating the operative results performed by analyzing the recovery rate using the Japanese Orthopaedic Association (JOA) scoring system.

**Results:**

Analysis were conducted on a total 20 patients undergoing surgery via anterior approach (9 patients), posterior approach with fixation (3 patients), and anterior. Posterior approach (8 patients). Two patients underwent surgery with hybrid decompression fixation technique. There were two patients who had decreased level of the JOA score which need posterior decompression. One patient had early onset post of infection in posterior approach.

**Conclusions:**

Our strategy base on naderi algorithm were effective and the follow up showed stable clinical results which depended on the completely removal of the possible causes of the pathology.

**Keywords:**

Cervical spine, Multilevel, Myelopathy


					Volume 4, Suppl. 1 Nov 2012
Publisher: Kermanshah University of Medical Sciences
URL: http://www.jivresearch.org
Copyright:
Congress of Iranian Neurosurgeons, 14-16 November 2012, Kermanshah, Iran
Paper No. 66
Surgical decompression strategies for treatment of multilevel
cervical myelopathy comparison of outcome with single level
cases
Misagh Shafizad a,*
, Guive sharifi a
a
Neurosurgery Department, Loghman Hakim Hospital, Shaheed Beheshti University of Medical Sciences, Tehran, Iran.
Abstract:
Background: Selecting the treatment of choice for multilevel cervical myelopathy remains a controversial issue. The
most important factor in choosing a treatment method for patients with cervical myelopathy is the sagittal curvature
and the site of compressive pathology.
Methods: A total of 20 cases were available for the last 4 years with acceptable follow-up (f/u) of at least one
year after surgery.Clinical examinations were obtained before surgery, 3 months post-surgery and at f/u.
Evaluating the operative results performed by analyzing the recovery rate using the Japanese Orthopaedic
Association (JOA) scoring system.
Results: Analysis were conducted on a total 20 patients undergoing surgery via anterior approach (9 patients),
posterior approach with fixation (3 patients), and anterior. Posterior approach (8 patients). Two patients underwent
surgery with hybrid decompression fixation technique. There were two patients who had decreased level of the JOA
score which need posterior decompression. One patient had early onset post of infection in posterior approach.
Conclusions: Our strategy base on naderi algorithm were effective and the follow up showed stable clinical results
which depended on the completely removal of the possible causes of the pathology.
Keywords:
Cervical spine, Multilevel, Myelopathy
* Corresponding Author at:
Misagh Shafizad: Neurosurgery Department, Loghman Hakim Hospital, Shaheed Beheshti University of Medical Sciences, Tehran, Iran.
Tel: 09111540432, Email: mishafizad@gmail.com, (Shafizad M.).

				

